# A New Single-Parameter Bees Algorithm

**DOI:** 10.3390/biomimetics9100634

**Published:** 2024-10-18

**Authors:** Hamid Furkan Suluova, Duc Truong Pham

**Affiliations:** Department of Mechanical Engineering, The University of Birmingham, Birmingham B15 2TT, UK; d.t.pham@bham.ac.uk

**Keywords:** Bees Algorithm, nature-inspired algorithm, bee-inspired algorithm, metaheuristics, continuous optimisation, combinatorial optimisation

## Abstract

Based on bee foraging behaviour, the Bees Algorithm (BA) is an optimisation metaheuristic algorithm which has found many applications in both the continuous and combinatorial domains. The original version of the Bees Algorithm has six user-selected parameters: the number of scout bees, the number of high-performing bees, the number of top-performing or “elite” bees, the number of forager bees following the elite bees, the number of forager bees recruited by the other high-performing bees, and the neighbourhood size. These parameters must be chosen with due care, as their values can impact the algorithm’s performance, particularly when the problem is complex. However, determining the optimum values for those parameters can be time-consuming for users who are not familiar with the algorithm. This paper presents BA_1_, a Bees Algorithm with just one parameter. BA_1_ eliminates the need to specify the numbers of high-performing and elite bees and other associated parameters. Instead, it uses incremental k-means clustering to divide the scout bees into groups. By reducing the required number of parameters, BA_1_ simplifies the tuning process and increases efficiency. BA_1_ has been evaluated on 23 benchmark functions in the continuous domain, followed by 12 problems from the TSPLIB in the combinatorial domain. The results show good performance against popular nature-inspired optimisation algorithms on the problems tested.

## 1. Introduction

Metaheuristics have attracted interest because of their ability to find near-optimal solutions through rapid iterative computations [1,2]. The effectiveness of exact methods decreases as the size and complexity of problems increase. Therefore, metaheuristics are more practical [3]. They are competent at addressing multi-objective optimisation challenges and perform well with limited data or computing resources. Metaheuristics are versatile and adjustable, making them effective for finding answers to a broad spectrum of optimisation problems across different sectors [4,5,6].

Metaheuristics provide advantages in solving intricate problems [7]. However, they have several limitations [8,9]. These include the risk of being trapped at a local optimum and leading to premature convergence, difficulties in selecting appropriate parameter configurations, causing poor performance, and the inability to guarantee that the solution found is optimal [9,10].

The number of initial parameters and their numerical values are important in finding near-optimal solutions because they affect various factors, such as the convergence rate, number of evaluations and exploration-exploitation balance. These factors are typically employed to assess the efficiency of a metaheuristic [11]. Additionally, novice researchers may be unsure about the parameter configuration for solving a particular problem. Changing the parameter settings might lead to different exploration and exploitation approaches, ultimately affecting the quality of the solutions achieved. Therefore, users must carefully select appropriate parameter values based on the problem to obtain the best results.

The Bees Algorithm (BA) is an intelligent computing method based on the foraging behaviour of honeybees [12]. It was developed in 2005 to solve continuous optimisation problems. Since its first introduction, the BA has attracted attention because of its versatility and ability to discover near-optimal solutions efficiently. The BA has also been used to solve several well-known combinatorial problems, including the travelling salesman problem (TSP), vehicle routing problem (VRP) and production planning and scheduling problems.

This paper introduces BA_1_, a single-parameter Bees Algorithm and evaluates its performance against those of different well-known algorithms. Parameter reduction is achieved via k-means clustering. Clustering is an unsupervised learning method which categorises unlabelled data by identifying their shared properties [13]. The k-means algorithm is a well-studied method for categorising the provided data items into k distinct clusters through an iterative process which converges to a local minimum [14]. There are various examples where metaheuristics are used to perform clustering as an optimisation problem. However, to the authors’ knowledge, employing clustering to enhance optimisation algorithms has not been studied.

Like metaheuristics, the standard k-means clustering algorithm requires selecting an appropriate parameter (the K value) to process the clustering. There are several techniques for managing the parameter configurations for k-means, one of which is incremental k-means, which was developed by Pham et al. [15]. This explores the total distortion amount for each K value by executing a cluster centre jump. Pham et al. [16] proposed a function which uses global distortion to evaluate clustering results. This function facilitates the automatic selection of the best number of clusters for the k-means method.

This paper is structured as follows. Section 2 introduces the Bees Algorithm and some of its recent variants. Section 3 explains the proposed single-parameter Bees Algorithm. Section 4 presents the results of evaluating this new algorithm against the Bat [17], Grey Wolf [18] and Whale [19] optimisation algorithms on 23 continuous benchmark functions and against the same three algorithms plus Moth Flame Optimisation [20] and Particle Swarm Optimisation [21] algorithms on 12 combinatorial problems from the TSPLIB. Section 5 concludes the paper.

## 2. The Bees Algorithm

Honeybees are social insects which have inspired researchers to use their behaviours, including food foraging, as the basis of numerous optimisation algorithms [22]. Bees in nature forage the fields surrounding their hive for food, looking for flower patches abundant in nectar. Upon reaching the hive, they deposit the collected nectar. Bees pass information about the food to the hive by dancing (i.e., waggle dancing) on a specific area of the hive called the “dance floor”. The waggle dance indicates the direction, distance and quality of the nectar supply. The number of recruited bees depends on the assessment of the patch’s quality. Food sources which are more abundant and have higher-quality nectar tend to attract more foragers, thus improving the food collection process [23].

The Bees Algorithm, which mimics this food-foraging behaviour, was first proposed by Pham et al. [24]. This version is known as the basic or original Bees Algorithm (BA_O_). In BA_O_, there are six user-selected parameters: the number of scout bees (*n*), the number of elite selected patches (*e*), the number of good selected patches (*m–e*), the number of recruited bees for the elite patches (nep), the number of recruited bees for the good patches (*nsp*) and the neighbourhood size (*ngh*). Although BA_O_ was introduced to address continuous optimisation, it has subsequently been applied to combinatorial problems such as scheduling [25] and PCB assembly [26]. Algorithm 1 is the pseudocode of the original BA.
**Algorithm 1:** Original Bees Algorithm1Start2Input the required parameters *n*, *e*, *m*, *nep*, *nsp*, *ngh*, *MaxIt*3Generate *n* initial solutions4Evaluate the fitness of the *n* initial solutions5Select the best m solution for neighbourhood search6
while iteration < *MaxIt* do7

for each site *i*, (*i* = 1, …, *e*) do8


Exploit site within *ngh* of the site with *nep* forager bees (Equation (3)) and Evaluate fitness9


if better solution found replace site10

end for11

for each site *j*, (*j* = *e* + 1, …, *m*) do12


Exploit site within *ngh* of the site with *nsp* forager bees (Equation (3)) and Evaluate fitness13


if better solution found replace site14

end for15

for each site *k*, (*k* = *m* + 1, …, *n*) do16


Explore site *n-m* scout bees (Equation (2)) and Evaluate fitness17

end for18
end while19Return the best-so-far solution

In the initialisation step of BA_O_, the user inputs the aforementioned parameters and the stopping criteria. The algorithm then starts the optimisation process by sending n scout bees (*X_s_*) to the search space with a uniform random distribution within its lower (*x_min_*) and upper (*x_max_*) boundaries (Equation (1)). Then, the algorithm evaluates the fitness of each scout bee. According to the quality of food sources, scout bees recruit forager bees (i.e., nep for the e elite patches and *nsp* for the (*m–e*) good selected patches) to exploit the source. These forager bees *X_f_* look for locations with better fitness scores for their patches, the initial size of which is defined by *ngh* (Equations (2) and (3)). The remaining (*n–m*) scout bees continue to explore the search space randomly. The algorithm updates the obtained solution at the end of each cycle and returns the best-so-far solution when a stopping criterion is met:

(1) $$X_{s}\left(i\right)=U\left[x_{min},\;x_{max}\right],\;\;\;\;\;\;\;\;\;\;\;\;\forall i=1,\;\cdots ,n,$$

(2) $$r=ngh\times \left(x_{min},\;x_{max}\right),$$

(3) $$X_{f}\left(i,j\right)=X_{s}\left(i\right)+U\left[-r,\;r\right],\;\;\;\;\;\;\;\forall i=1,\cdots ,\;n;\;j=\left\{\begin{array}{c} 1,\cdots ,nep\;\;\;\;\;\;\;\;\;\;\;\;\;\;\;\;if\;i\leq e\\ 1,\cdots ,nsp\;\;\;\;\;\;\;if\;e<i\leq m \end{array}\right..$$

The shrinking strategy, which reduces the patch size when the algorithm is not able to improve the best solution on the patch, was employed to obtain a more exploitative local search and increase the density around a local optimum [27]. Pham and Darwish [28] also utilised this method in addition to fuzzy selection of the flower patches. Parameter reduction was performed via the fuzzy Bees Algorithm (BA_Fuzzy_), which uses fuzzy logic to choose elite patches among the selected patches and the foragers to perform a local search.

An improved version of the BA, referred to as the standard Bees Algorithm (BA_S_), which includes shrinking and site abandonment, was introduced in [23]. With site abandonment, the bees leave a patch when the situation is stagnant (i.e., there is no improvement following a set number of trials during a local search). It is a method complementary to the shrinking strategy since they both act on a lack of improvement in the patch’s best solution. While shrinking improves the exploitation capability of the algorithm, site abandonment helps to avoid local optima.

BA_S_ becomes an eight-parameter metaheuristic with the addition of the shrinking rate (*shrink*) and stagnation limit (*stlim*) to the existing parameters of BA_O_ (i.e., *n*, *e*, *m*, *nep*, *nsp* and *ngh*). Here, *shrink* is a positive real multiplicative factor less than or equal to one to be applied to *ngh*, while *stlim* is a positive integer and is the number of trials made before a patch is abandoned.

**Figure 1:**
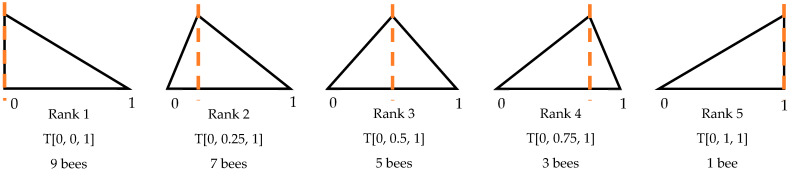
Triangular distribution for *n* = 5 and *nep* = 9. From left to right: the highest-ranked to the lowest-ranked patches. More foragers (9 bees) were recruited to the highest-ranked patch than the other patches. The lowest-ranked patch received the smallest number of foragers (1 bee). (The orange lines indicate the peaks of the triangles).

Ismail et al. [29] achieved parameter reduction with BA_2_, a two-parameter version of the BA, for both continuous and combinatorial problems. The reduction is performed by combining the exploration and exploitation phases while maintaining the core principles of the BA. BA_2_ only requires the user to define the number of patches (*n*) and the maximum number of foragers sent to the top-ranked (elite) patch (*nep*). The number of foragers for other patches is determined via Equation (4), where $w_{max}$ represents *nep*, $w_{min}$ equals one, and *k* is the rank of a patch (i.e., *k* = 1 for the best patch, e.g., Figure 1). BA_2_ employs a triangular distribution to place foragers on the patches. The centre of a patch corresponds to the summit of its triangular distribution. There is no crisply defined neighbourhood which now covers the whole search space [29]:

(4) $$w_{k}=w_{max}+\left(k-1\right)\left(\frac{w_{min}-w_{max}}{n-1}\right).$$

The initial version of BA_2_ did not have a shrinking rate or stagnation limit (i.e., BA_2_ is a two-parameter version of BA_O_). If the shrinking rate and stagnation limit are included, then the total number of parameters is four, and the algorithm becomes a reduced-parameter version of BA_S_ [30].

Hartono and Pham [31] introduced the Fibonacci Bees Algorithm (BA_F_), which reduces parameters via the Fibonacci sequence. BA_F_ discards two parameters from BA_S_, namely the number of elite sites (*e*) and the number of recruited bees for elite sites (*nep*). The *m* selected sites are ranked, and the number of recruited bees for each selected site is determined according to its rank using the Fibonacci sequence.

Suluova, Hartono and Pham [22] proposed a continuous version of BA_F_ with a total of five parameters: the number of scout bees (*n*), the number of selected sites (*m*), the maximum number of foragers for the top-ranked site (*nr*), the shrinking rate (*shrink*) and the stagnation limit (*stlim*). Their results showed that BA_F_ is competitive and has an improved success rate for some benchmark functions compared with BA_S_.

## 3. Details of BA_1_

The k-means clustering method is employed to determine the number of patches by grouping the search agents (i.e., the bees). Grouping individuals in the search space eliminates the need for parameters related to the distribution of bees to sites, such as the number of elite patches (*e*), the number of recruited bees for elite patches (*nep*), the number of selected patches (*m*), and the number of recruited bees for other selected patches (*nsp*). As mentioned above, to group the bees in the search space without increasing the number of parameters, the cluster selection function *f*(*K*) by Pham et al. [16] is used automatically to select the optimal number of clusters. As explained in [16], in the first step, to determine an appropriate K value, the distortions of all the clusters are calculated separately for each K value (Equation (5)). Then, the total distortion of the clusters is obtained via Equation (6):

(5) $$I_{Kj}=\sum_{t=1}^{N_{j}}\left[d(x_{jt},\;w_{j}\right){]}^{2},$$

(6) $$S_{K}=\overset{K}{\underset{j=1}{\sum }}I_{Kj}.$$

In Equation (5), $I_{Kj}$ represents the distortion of cluster j when the bees form *K* clusters, $N_{j}$ is the number of bees belonging to cluster *j*, $x_{jt}$ is the *t*^th^ element, and $d\left(x_{jt},\;w_{j}\right)$ is the distance between $x_{jt}$ and the centre $w_{j}$ of cluster *j*. $S_{K}$ is the actual distortion for a specified *K* value, whereas $\alpha_{K}\;S_{K-1}$ estimates the distortion for *K* based on the actual distortion for (*K* − 1). BA_1_ keeps a record of the total distortions and determines the optimum *K* value using $f\left(K\right)$ (Equation (7)) for the initialised bee distribution. If the distribution is uniform (i.e., the whole population forms a single cluster), then $f\left(K\right)$ becomes one. However, if the bees in the search space are dense at some locations, then $f\left(K\right)$ decreases, and this confirms that there are some well-defined clusters. In the equations below, $\alpha_{K}$ is a weight factor, while $N_{d}$ is the number of dimensions:

(7) $$f\left(K\right)=\left\{\begin{array}{c} 1\\ \frac{S_{K}}{\alpha_{K}\;S_{K-1}}\\ 1 \end{array}\;\;\;\;\;\;\;\;\begin{array}{c} if\;K=1,\\ if\;S_{K-1}\neq 0,\;\forall K>1,\\ if\;S_{K-1}=0,\;\forall K>1, \end{array}\right.$$

(8) $$\alpha_{K}=\left\{\begin{array}{c} 1-\frac{3}{4N_{d}}\\ \alpha_{K-1}+\frac{1-\alpha_{K-1}}{6} \end{array}\;\;\;\begin{array}{c} if\;K=2\;and\;N_{d}>1,\\ if\;K>2\;and\;N_{d}>1. \end{array}\right.$$

Using the incremental k-means clustering method [15] and the *K* selection function [16], the total population of bees in the search space is categorised into one or more groups. As a first step in clustering, the entire population of n scout bees (*X_s_*) explores the search space (Equation (9)). Then, the algorithm measures the Euclidean distances *d* between the fittest bee (*X_s_*(1)) and other individuals (Equation (10)). With the incremental k-means approach, using these distances, the algorithm chooses the most appropriate *k* value for the population and the problem. The selected *k* value (i.e., the number of clusters) becomes the number of patches, and the individuals belonging to each cluster turn into forager bees to exploit the sites for which the centre is the best bee in the cluster. As a result of this process, the proposed algorithm eliminates the need to set the numbers of elite and good patches and the number of bees recruited for these patches:

(9) $$X_{s}\left(i\right)=U\left[x_{min},{\;x}_{max}\right],\;\;\;\;\;\;\;\;\;\;\;\;\forall i=1,\;\cdots ,n,$$

(10) $$d\left(i\right)=\sqrt{{\left(X_{s}\left(1\right)\right)}^{2}-{\left(X_{s}\left(i\right)\right)}^{2}},\;\;\;\;\;\;\;\;\;\;\;\;\forall i=1,\;\cdots ,n.$$

**Figure 2:**
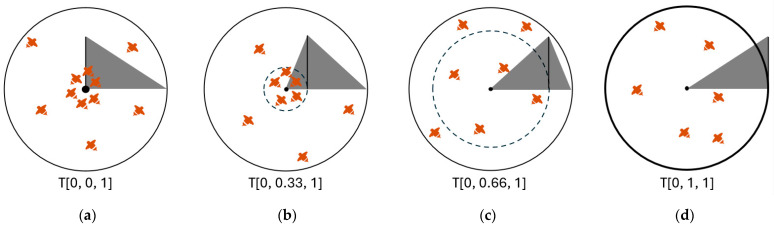
Example of swarming points for patches when *K* = 4 (**a**,**b**) bees swarm near the centre of highly ranked patches. (**c**,**d**) Bees are dispersed in lower-ranked patches.

The triangular probability distribution (*T* [0, *p_k_*, 1]) is employed to integrate exploration and exploitation. Here, *p_k_* represents the peak point of a patch and is determined using Equation (11), where k is the rank number of a patch (i.e., *k* = 1 is the best patch) and *K* is the total number of patches. A random number generator based on a triangular distribution (TRNG) generates numbers between 0 and 1 to determine a swarm radius *r* for each forager bee *j* on patch *k* (Equation (12)), where zero corresponds to the patch centre and one represents the furthest point from the centre. Then, a new solution *X_f_* is generated via Equation (13) within patch k, the centre of which is located at *X_s_*. The forager bees tend to exploit the patch when the rank is high. As can be seen in Figure 2, the majority of the foragers swarm near the centres of highly ranked patches. As a result of using a TRNG, the *ngh* parameter of the original BA is eliminated when the exploration (0.5 < *p_k_* < 1) and exploitation (0 < *p_k_* < 0.5) phases are merged:

(11) $$p_{k}=\frac{k-1}{K-1},\;\;\;\forall k=1,\cdots ,K.$$

(12) $$r\left(k,\;j\right)=ngh_{k}\times \left(x_{min},{\;x}_{max}\right)=T\left[0,\;p_{k},\;1\right]\times \left(x_{min},{\;x}_{max}\right).$$

(13) $$X_{f}\left(k,j\left(k\right)\right)=X_{s}\left(k\right)+U\left[-r\left(k,j\left(k\right)\right),r\left(k,j\left(k\right)\right)\right],\;\forall k=1,\cdots ,K;j\left(k\right)=\left\{\begin{array}{c} 1,\cdots ,w_{1}\;\;\;if\;k=1\\ \vdots \\ 1,\;\cdots ,\;w_{K}\;\;if\;k=K \end{array}\right.$$

It is known that the efficiency of BA increases with the shrinking and site abandonment strategies. However, determining the correct parameter values for those strategies can be challenging. Selecting an improper shrinking rate could cause the algorithm to miss an optimum and converge prematurely. Similarly, it is critical to select a suitable stagnation limit (i.e., the number of consecutive visits to a patch without improvements), since an incorrect limit could cause either early abandonment or not leaving the patch even if it has run out of nectar. Thus, although these two strategies can increase the algorithm’s performance, having to set two additional parameters makes it more complex for users. Thus, in BA_1_, the shrinking rate (*shrink*) is automatically determined by the algorithm itself based on the iteration number (Equation (14)). In this equation, the shrinking rate is adjusted between 0.75 and 1, decreasing from 1 in the first iteration to 0.75 in the last iteration. This causes a reduction in the patch size, which helps BA_1_ increasingly focus on exploitation towards the end. Furthermore, the stagnation limit (*stlim*) becomes dependent on the average bee number per site. After the algorithm determines the number of patches, *stlim* is calculated using Equation (15):

(14) $$shrink=1-\left(\frac{3}{4}\frac{it}{MaxIt}\right),\;\;\;\;\forall it=1,\ldots ,\;MaxIt,$$

(15) $$stlim=\frac{ColonySize}{K}.$$

**Figure 3:**
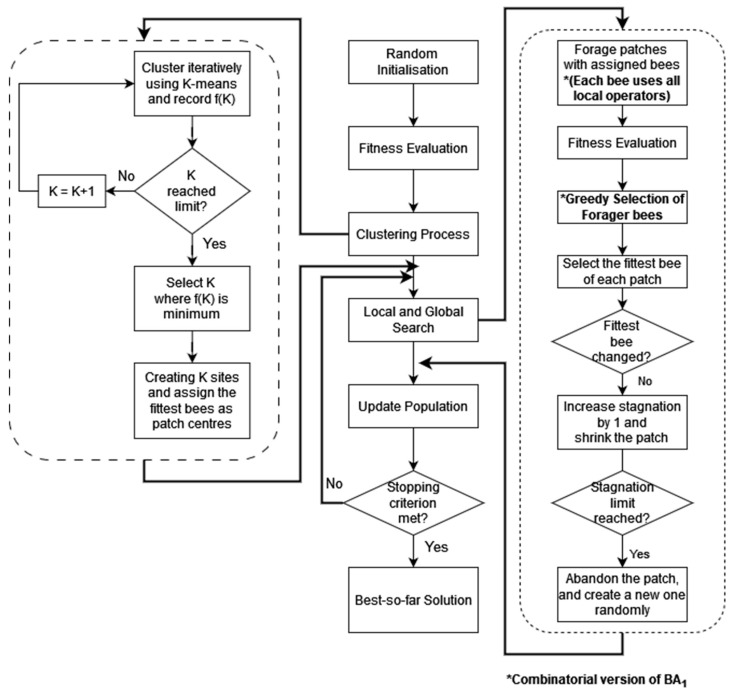
Flowchart for BA_1_.

Figure 3 shows the general flowchart for BA_1_ which has been designed for both continuous and combinatorial optimisation problems. There are two minor differences between the continuous and combinatorial versions of BA_1_, namely the distance metric and the local operators used. In this work, the Hamming distance was adopted as the distance metric for combinatorial problems including vehicle routing, sequence planning and production scheduling. For those problems, the Euclidean distance ubiquitous in continuous optimisation cannot be employed.

The main local search operator for the continuous optimisation version is mutation, although other operators such as creep and randomisation can also be used. As with other combinatorial variants of the BA, the swap, insertion and reversion local operators are employed to generate new solutions. However, while previous variants only used one local operator to produce one solution from an existing solution in each iteration, BA_1_ applies all local operators and selects the fittest one among the solutions created. This greedy selection increases the number of fitness evaluations per cycle but helps the process converge to near-optimal solutions faster by leveraging the strengths of the different operators.

## 4. Experiments and Results

Experiments to assess the performance of BA_1_ were performed on both continuous and combinatorial problems. Twenty-three benchmark functions were used to evaluate BA_1_ in the continuous domain. The performance of BA_1_ was compared with that of the bat algorithm (BAT) [17], Grey Wolf Optimiser (GWO) [18] and Whale Optimisation Algorithm (WOA) [19]. For the combinatorial domain, BA_1_ was applied to 12 datasets from the Travelling Salesman Problem Library (TSPLIB) [32] and compared with BAT [17], the Discrete Whale Optimisation Algorithm (DWOA) [19], Grey Wolf Optimiser (GWO) [18], Moth Flame Optimisation (MFO) [20] and Particle Swarm Optimisation (PSO) [21]. The comparator algorithms were chosen for their proven competitive performance [33].

Table 1 lists the details of the functions used for continuous optimisation. The first six functions (F1–F6) were used to evaluate the exploitation ability of the algorithms. These functions had 30 dimensions and large domains. F6–F13 were 30 dimensional problems for testing the algorithms’ exploration performance. Functions F14–F23 were used to demonstrate the algorithms’ ability to handle low-dimensional problems where the global optima were not zero.

The values of the parameters for the different algorithms are given in Table 2. The one parameter for BA_1_, which is the total number of bees, was set at 100, equal to the number of search agents for all of the comparator algorithms. The other parameters of the comparator algorithms were taken from the literature [34]. Fifty independent runs of each algorithm were conducted per function. The stopping criterion was 500,000 fitness evaluations. If the obtained result was less than 10^−10^, then it was taken to be “0”. Table 3 presents the results of the continuous optimisation experiments.

From Table 3, it can be noted that BA_1_ was the lone top performer for 12 benchmark problems (F5, F6, F11, F12, F13, F14, F16, F18, F20, F21, F22 and F23) and shared the first place with others for four problems (F1, F2, F10 and F17). BA_1_ yielded strong results for both low- and high-dimensional functions, showing no search bias towards the origin. BA_1_ obtained the exact solutions for eight functions (F1, F2, F6, F10, F12, F13, F16 and F22) with a null standard deviation, which reflects its precision in reaching the global optimum. In addition, the proposed algorithm exhibited a high degree of robustness, generally finding stable solutions with low variability. Although BA_1_ was not the best performer for some problems, it still provided competitive solutions with low standard deviations, indicating its ability to explore and exploit. Thus, it can be stated that BA_1_ demonstrated an excellent performance through numerous trials and consistently provided precise and reliable solutions in the continuous domain.

Table 4 shows the results for the 12 combinatorial problems selected from the TSPLIB. The results for BAT, DWOA, GWO, MFO and PSO were taken from the study by Zhang et al. [35]. BA_1_ was again run 50 times, with each run limited to 1000 iterations. The population size was set equal to the number of cities in each problem. The comparison measure was the error rate (ER), which is the difference between the best-obtained solution (BOS) and the best-known solution (BKS) (Equation (16)):

(16) $$ER=\frac{BOS-BKS}{BKS}\times 100.$$

From Table 4, it can be noted that BA_1_ had the lowest ER in eight of the problems, namely Ch150, D198, Eil51, Fl417, KroA100, Pr76, St70 and Tsp225. Only for one dataset (Oliver30) did BA_1_ have the poorest ER, sharing the bottom rank with PSO. BA_1_ showed higher performance when the complexity of the problem increased. Excluding Pr107, BA_1_ was consistently the top performer for problems involving more than 100 cities. Although it was not the best algorithm for some problems (Oliver30, Berlin52 and Eil76), it was competitive and able to provide near-optimal solutions. The performance of BA_1_ was above those of the comparators when considering the average ER of 12 datasets. When taking the average ER over all 12 problems, BA_1_ was in first place with an average ER of 5.27%, followed by DWOA (6.58%), BAT (8.24%), MFO (8.52%), GWO (9.08%) and PSO (14.40%).

## 5. Conclusions

This paper presented BA_1_, a single-parameter BA for both continuous and combinatorial optimisation. BA_1_ only requires the user to set the size of the bee population. BA_1_ starts by randomly generating initial solutions in the search space. Next, BA_1_ employs the incremental k-means method to cluster bees based on their distances to the fittest bee. The algorithm iteratively processes clustering and keeps a memory of the total distortion. Then, it selects the best *K* value for which the distortion function *f*(*K*) is minimal. The bees are clustered into patches and forage the patch to which they belong. Clustering automatically gives the number of bees for each patch. As in BA_2_, exploration and exploitation are merged via a probability distribution of foragers which concentrates bees around the top solutions and disperses them more randomly across the search space in the case of lower-ranked solutions. Additionally, shrinking and site abandonment follow preset strategies (i.e., shrinking is iteration-dependent, and site abandonment is a function of the number of patches). Therefore, the number of parameters decreases from six or eight (original BA: *n*, *e*, *m*, *nep*, *nsp*, *ngh*; standard BA: original BA parameters plus *shrink* and *stlim*) to one (*n*). BA_1_ simplifies the parameter configuration by minimising the number of parameters, making it even easier to use than BA_2_. The performance of BA_1_ was evaluated on 23 benchmark functions for continuous optimisation and 12 datasets from the TSPLIB for combinatorial optimisation and compared with those of popular metaheuristics. The results show that BA_1_ performed well against the comparator algorithms. BA_1_ demonstrated a high degree of reliability across numerous problems of varying complexity and size. Future work will extend the comparison to a larger set of benchmarks including standard engineering test problems and, in recognition of the no free lunch theory [36], seek to define the classes of problems to which BA_1_ is best suited.

## Figures and Tables

**Table 1:** Details of the benchmark functions.

Functions	Dim	Bounds	Global Optimum
$F_{1}\left(\vec{x}\right)=\sum_{i=1}^{n}{x_{i}}^{2}\;$	30	[−100, 100]	$F_{1}\left(\vec{x}\right)=0$
$F_{2}\left(\vec{x}\right)=\sum_{i=1}^{n}\left|x_{i}\right|+\prod_{i=1}^{n}x_{i}$	30	[−100, 100]	$F_{2}\left(\vec{x}\right)=0$
$F_{3}\left(\vec{x}\right)=\sum_{i=1}^{n}{\left(\sum_{j=1}^{n}x_{j}\right)}^{2}$	30	[−100, 100]	$F_{3}\left(\vec{x}\right)=0$
$F_{4}\left(\vec{x}\right)=\max\left(\left|x_{i}\right|,\;1\leq i\leq n\right)$	30	[−100, 100]	$F_{4}\left(\vec{x}\right)=0$
$F_{5}\left(\vec{x}\right)=\sum_{i=1}^{n-1}\left(100{\left(-x_{i}^{2}+x_{i+1}\right)}^{2}+{\left(x_{i}-1\right)}^{2}\right)$	30	[−30, 30]	$F_{5}\left(\vec{x}\right)=0$
$F_{6}\left(\vec{x}\right)=\sum_{i=1}^{n-1}{\left(x_{i}+0.5\right)}^{2}$	30	[−100, 100]	$F_{6}\left(\vec{x}\right)=0$
$F_{7}\left(\vec{x}\right)=\sum_{i=1}^{n}ix_{i}^{4}+rand\left[0,\;1\right]$	30	[−1.28, 1.28]	$F_{7}\left(\vec{x}\right)=0$
$F_{8}\left(\vec{x}\right)=\sum_{i=1}^{n-1}-x_{i}\sin\left(\sqrt{\left|x_{i}\right|}\right)$	30	[−500, 500]	$F_{8}\left(\vec{x}\right)=-418.98\times n$
$F_{9}\left(\vec{x}\right)=\sum_{i=1}^{n}\left(x_{i}^{2}-10\cos\left(2\pi x_{i}\right)+10\right)$	30	[−5.12, 5.12]	$F_{9}\left(\vec{x}\right)=0$
$F_{10}\left(\vec{x}\right)=-20\exp\left(-0.2\sqrt{\left(\frac{1}{n}\right)\sum_{i=1}^{n}x_{i}^{2}}\right)-\exp\left(\frac{1}{n}\sum_{i=1}^{n}\cos\left(2\pi x_{i}\right)\right)+20+e$	30	[−32, 32]	$F_{10}\left(\vec{x}\right)=0$
$F_{11}\left(\vec{x}\right)=\left(\vec{x}\right)=\frac{1}{4000}\sum_{i=1}^{n}{\left(x_{i}\right)}^{2}-\prod_{i=1}^{n}\cos\left(\frac{x_{i}}{\sqrt{i+1}}\right)+1$	30	[−600, 600]	$F_{11}\left(\vec{x}\right)=0$
$F_{12}\left(\vec{x}\right)=\frac{\pi }{n}\left\{10\sin\left(\pi y_{1}\right)+\sum_{i=1}^{n-1}{\left(y_{i}-1\right)}^{2}\left[1+10\sin^{2}\left(\pi y_{i+1}\right)\right]+{\left(y_{n}-1\right)}^{2}\right\}+\sum_{i=1}^{n}u\left(x_{i},\;10,\;100,\;4\right)$	30	[−50, 50]	$F_{12}\left(\vec{x}\right)=0$
$F_{13}\left(\vec{x}\right)=0.1\left\{\sin^{2}\left(3\pi x_{1}\right)+\sum_{i=1}^{n-1}{\left(x_{i}-1\right)}^{2}\left[1+\sin^{2}\left(3\pi x_{i}+1\right)\right]+{\left(x_{n}-1\right)}^{2}\left[1+\sin^{2}\left(2\pi x_{n}\right)\right]\right\}+\sum_{i=1}^{n}u\left(x_{i},\;5,\;100,\;4\right)$	30	[−50, 50]	$F_{13}\left(\vec{x}\right)=0$
$F_{14}\left(\vec{x}\right)={\left(\frac{1}{500}+\sum_{j=1}^{25}\frac{1}{j+\sum_{i=1}^{2}{\left(x_{i}-a_{ij}\right)}^{6}}\right)}^{-1}$	2	[−65, 65]	$F_{14}\left(\vec{x}\right)=1$
$F_{15}\left(\vec{x}\right)=\sum_{i=1}^{11}{\left[a_{i}-\frac{x_{1}\left({b_{i}}^{2}+b_{i}x_{2}\right)}{{b_{i}}^{2}+b_{i}x_{3}+x_{4}}\right]}^{2}$	4	[−5, 5]	$F_{15}\left(\vec{x}\right)=0.00030$
$F_{16}\left(\vec{x}\right)=4x_{1}^{2}-2.1x_{1}^{4}+\frac{1}{3}x_{1}^{6}+x_{1}x_{2}-4x_{2}^{2}+4x_{2}^{4}$	2	[−5, 5]	$F_{16}\left(\vec{x}\right)=-1.0316$
$F_{17}\left(\vec{x}\right)={\left(-\frac{5.1}{4\pi^{2}}x_{1}^{2}+\frac{5}{\pi }x_{1}-6\right)}^{2}+10\left(1-\frac{1}{8\pi }\right)cosx_{1}+10$	2	[−5, 5]	$F_{17}\left(\vec{x}\right)=0.398$
$F_{18}\left(\vec{x}\right)=\left[1+{\left(x_{1}+x_{2}+1\right)}^{2}\left(19-14x_{1}+3x_{1}^{2}-14x_{2}+6x_{1}x_{2}+3x_{2}^{2}\right)]\times [30+{\left(2x_{1}-3x_{2}\right)}^{2}\left(18-32x_{1}+12x_{1}^{2}+48x_{2}-36x_{1}x_{2}+27x_{2}^{2}\right)\right]$	2	[−2, 2]	$F_{18}\left(\vec{x}\right)=3$
$F_{19}\left(\vec{x}\right)=-\sum_{i=1}^{4}c_{i}\exp\left(-\sum_{j=1}^{3}a_{ij}{\left(x_{j}-p_{ij}\right)}^{2}\right)$	3	[1, 3]	$F_{19}\left(\vec{x}\right)=-3.86$
$F_{20}\left(\vec{x}\right)=-\sum_{i=1}^{4}c_{i}\exp\left(-\sum_{j=1}^{6}a_{ij}{\left(x_{j}-p_{ij}\right)}^{2}\right)$	6	[0, 1]	$F_{20}\left(\vec{x}\right)=-3.32$
$F_{21}\left(\vec{x}\right)=-\sum_{i=1}^{5}{\left[c_{i}+{\left(x-a_{i}\right)}^{T}\left(x-a_{i}\right)\right]}^{-1}$	4	[0, 10]	$F_{21}\left(\vec{x}\right)=-10.1532$
$F_{22}\left(\vec{x}\right)=-\sum_{i=1}^{7}{\left[c_{i}+{\left(x-a_{i}\right)}^{T}\left(x-a_{i}\right)\right]}^{-1}$	4	[0, 10]	$F_{22}\left(\vec{x}\right)=-10.4028$
$F_{23}\left(\vec{x}\right)=-\sum_{i=1}^{10}{\left[c_{i}+{\left(x-a_{i}\right)}^{T}\left(x-a_{i}\right)\right]}^{-1}$	4	[0, 10]	$F_{23}\left(\vec{x}\right)=-10.5363$
$u=\left(x_{i},a,k,m\right)=\left\{\begin{array}{c} k{\left(x_{i}-a\right)}^{m}\;,\;\;and\;x_{i}>a\\ 0\;,\;\;and-a<x_{i}<a\\ k{\left(-x_{i}-a\right)}^{m}\;,\;\;and\;x_{i}<-a\; \end{array}\right.$	-	-	-

**Table 2:** Parameter settings used in the experiments.

	BA_1_	BAT	GWO	WOA
Total Population	100	100	100	100
Loudness	NA	1	NA	NA
Pulse Rate	NA	1	NA	NA
Alpha	NA	0.97	NA	NA
Gamma	NA	0.1	NA	NA
Minimum Frequency	NA	0	NA	NA
Maximum Frequency	NA	2	NA	NA

NA: Not Applicable

**Table 3:** Results of continuous optimisation experiments.

Functions	BA_1_		BAT		GWO		WOA	
	Mean	Std Dev	Mean	Std Dev	Mean	Std Dev	Mean	Std Dev
F1	**0.00**	0.00	1.43 × 10^4^	3.07 × 10^3^	**0.00**	0.00	**0.00**	0.00
F2	**0.00**	0.00	1.15 × 10^3^	1.02 × 10^2^	**0.00**	0.00	**0.00**	0.00
F3	1.57 × 10^−2^	1.72 × 10^−2^	1.94 × 10^4^	5.39 × 10^3^	**0.00**	0.00	6.75	9.04
F4	3.91	3.54	6.06 × 10^1^	4.65	**0.00**	0.00	1.62	5.43
F5	**2.71**	4.38	1.61 × 10^2^	2.63 × 10^2^	2.82 × 10^1^	1.03	2.37 × 10^1^	2.06 × 10^−1^
F6	**0.00**	0.00	1.47 × 10^4^	2.71 × 10^3^	3.50	6.21 × 10^−1^	7.90 × 10^−7^	3.12 × 10^−7^
F7	4.87 × 10^−2^	1.99 × 10^−2^	2.97 × 10^−2^	1.24 × 10^−2^	4.16 × 10^−4^	1.01 × 10^−4^	**9.59 × 10^−5^**	1.07 × 10^−4^
F8	−1.14 × 10^4^	1.79 × 10^2^	−6.03 × 10^3^	5.84 × 10^2^	−5.93 × 10^3^	7.84 × 10^2^	**−1.24 × 10^4^**	4.37 × 10^2^
F9	1.99 × 10^−2^	1.39 × 10^−1^	1.78 × 10^2^	2.69 × 10^1^	2.44 × 10^1^	4.03	**0.00**	0.00
F10	**0.00**	0.00	1.90 × 10^1^	2.23 × 10^−1^	2.05	1.37	**0.00**	0.00
F11	**1.48 × 10^−4^**	1.04 × 10^−3^	4.68 × 10^2^	4.27 × 10^1^	6.14 × 10^−3^	4.12 × 10^−3^	4.03 × 10^−4^	2.03 × 10^−3^
F12	**0.00**	0.00	3.44 × 10^1^	9.20	1.06	6.42 × 10^−1^	1.44 × 10^−7^	6.22 × 10^−8^
F13	**0.00**	0.00	1.03 × 10^2^	9.52	2.09	4.98 × 10^−1^	2.68 × 10^−6^	2.58 × 10^−6^
F14	**9.98 × 10^−1^**	3.33 × 10^−16^	9.53	7.53	7.16	4.89	9.98 × 10^−1^	4.66 × 10^−15^
F15	6.68 × 10^−4^	2.10 × 10^−3^	1.34 × 10^−3^	1.66 × 10^−3^	4.04 × 10^−3^	7.71 × 10^−3^	**4.19 × 10^−4^**	2.97 × 10^−4^
F16	**−1.03**	0.00	−9.99 × 10^−1^	1.60 × 10^−1^	−1.03	1.42 × 10^−11^	−1.03	1.96 × 10^−15^
F17	**3.98 × 10^−1^**	1.67 × 10^−16^	**3.98 × 10^−1^**	1.67 × 10^−16^	3.98 × 10^−1^	2.10 × 10^−9^	3.98 × 10^−1^	1.83 × 10^−11^
F18	**3.00**	2.66 × 10^−15^	7.32	9.90	3.00	7.34 × 10^−8^	3.00	9.98 × 10^−10^
F19	−3.00 × 10^−1^	2.78 × 10^−16^	**−3.86**	0.00	−3.00 × 10^−1^	2.78 × 10^−16^	−3.00 × 10^−1^	2.78 × 10^−16^
F20	**−3.32**	3.11 × 10^−15^	−3.26	5.94 × 10^−2^	−3.27	5.87 × 10^−2^	−3.25	6.49 × 10^−2^
F21	**−1.02 × 10^1^**	7.11 × 10^−15^	−5.48	3.08	−8.45	2.94	−1.02 × 10^1^	1.72 × 10^−7^
F22	**−1.04 × 10^1^**	0.00	−5.41	3.37	−9.68	2.01	−1.04 × 10^1^	9.04 × 10^−8^
F23	**−1.05 × 10^1^**	1.74 × 10^−14^	−5.78	3.53	−9.79	2.27	−1.05 × 10^1^	8.16 × 10^−8^

The best solutions are in **bold**. If two algorithms found the same solution, then the one with the smaller standard deviation was accepted as the best one. If the standard deviations were also the same, then both of them were accepted as the best one.

**Table 4:** Results of combinatorial experiments.

Problem (BKS)	BA_1_		BAT		DWOA		GWO		MFO		PSO	
	Mean	ER (%)	Mean	ER (%)	Mean	ER (%)	Mean	ER (%)	Mean	ER (%)	Mean	ER (%)
Berlin52 (7542)	7930	5.14	**7694**	**2.02**	7727	2.45	7898	4.72	8184	8.51	7862	4.24
Ch150 (6528)	**6928**	**6.13**	7440	13.97	7329	12.27	7384	13.11	7329	12.27	7833	19.99
D198 (15,780)	**16,240**	**2.92**	16,849	6.77	16,603	5.22	17,109	8.42	16,911	7.17	18,130	14.89
Eil51 (426)	**437**	**2.58**	439	3.05	445	4.46	441	3.52	449	5.4	445	4.46
Eil76 (538)	562	4.46	**561**	**4.28**	579	7.62	565	5.02	577	7.25	595	10.59
Fl417 (11,861)	**13,099**	**10.44**	15,532	30.95	13,886	17.07	15,492	30.61	14,087	18.77	18,688	57.56
KroA100 (21,282)	**22,018**	**3.46**	23,424	10.06	22,471	5.59	22,963	7.9	23,456	10.22	23,480	10.33
Oliver30 (420)	424	0.95	**420**	**0**	**420**	**0**	422	0.48	423	0.71	424	0.95
Pr76 (108,159)	**111,410**	**3.01**	111,989	3.54	111,511	3.1	114,261	5.64	114,377	5.75	115,265	6.57
Pr107 (44,303)	50,514	14.02	46,419	4.78	**45,780**	**3.33**	46,083	4.02	47,437	7.07	46,919	5.9
St70 (675)	**696**	**3.11**	718	6.37	712	5.48	726	7.56	710	5.19	732	8.44
Tsp225 (3916)	**4192**	**7.05**	4427	13.05	4399	12.33	4620	17.98	4469	14.12	5049	28.93

The best solutions are in **bold**.

## Data Availability

The source code for the proposed algorithm is accessible at https://github.com/hfsuluova/Single-Parameter_BeesAlgorithm (accessed on 8 October 2024).
